# Apparent Diffusion Coefficient Is a Novel Imaging Biomarker of Myopathic Changes in Liver Cirrhosis

**DOI:** 10.3390/jcm7100359

**Published:** 2018-10-15

**Authors:** Alexey Surov, Lisa Paul, Hans Jonas Meyer, Stefan Schob, Cornelius Engelmann, Andreas Wienke

**Affiliations:** 1 Department of Diagnostic and Interventional Radiology, University Hospital Leipzig, Liebigstrasse 20, 04103 Leipzig, Germany; lisa.paul@outlook.com (L.P.); jonas90.meyer@web.de (H.J.M.); stefan.schob@medizin.uni-leipzig.de (S.S.); 2 Section Hepatology, Department of Gastroenterology and Rheumatology, University Hospital Leipzig, Liebigstrasse 20, 04103 Leipzig, Germany; cornelius.engelmann@medizin.uni-leipzig.de; 3 Institute for Liver and Digestive Health, University College London, Royal Free Campus, Rowland Hill Street, London NW3 2PF, UK; 4 Institute of Medical Epidemiology, Biostatistics, and Informatics, Martin-Luther University, 06112 Halle-Wittenberg, Germany; andreas.wienke@uk-halle.de

**Keywords:** liver cirrhosis, sarcopenia, diffusion weighted imaging, apparent diffusion coefficient

## Abstract

Diffusion weighted imaging can provide information regarding tissue composition and can quantitatively characterize different pathological changes by means of apparent diffusion coefficient (ADC). The study comprised of 114 patients with liver cirrhosis—22 women and 92 men with a mean age of 56.5 ± 9.0 years. In all patients, the Model for End Stage-Liver Disease (MELD) score was calculated. Furthermore, 12 healthy persons (5 women, 7 men), mean age, 42.1 ± 16.2 years, were investigated as a control group. In all cases, magnetic resonance imaging of the liver/trunk was performed using different 3T scanners and diffusion weighted images were obtained with a multi-shot SE-EPI sequence. In all cases, polygonal regions of interest were manually drawn on the ADC maps along the contours of the iliopsoas and paravertebral muscles. The comparison of ADC values in groups was performed by Mann-Whitney-U tests. The association between ADC and MELD score was calculated by Spearman’s rank correlation coefficient. ADC values of the skeletal musculature were statistically much higher in comparison to those in the control group: 1.85 ± 0.46 × 10^−3^ mm^2^ s^−1^ vs. 1.23 ± 0.12 × 10^−3^ mm^2^ s^−1^, *p* = 0.001. ADC values showed statistically significant correlation with the MELD score (*r* = 0.473, *p* = 0.0001). Furthermore, ADC values differed between the subgroups with different values of the MELD score. ADC values correlated slightly with lactate dehydrogenase (LDH) (*r* = 0.381, *p* = 0.0001) and tended to correlate with C-reactive protein (CRP) (*r* = 0.171, *p* = 0.07) and alanine aminotransferase (ALAT) (*r* = −0.167, *p *= 0.076). ADC can reflect muscle changes in liver cirrhosis and shows statistically significant correlation with the MELD score. Therefore, ADC can be used as an imaging biomarker of myopathic changes in liver cirrhosis.

## 1. Introduction

Liver cirrhosis is the result of the progression of many forms of necrotic and inflammatory liver diseases and includes liver fibrosis, vascular remodeling, portal hypertension and liver failure [1].

According to the literature, liver cirrhosis is often accompanied with skeletal muscle loss or sarcopenia [2,3]. The pathogenesis of sarcopenia in liver cirrhosis is multifactorial. Malnutrition, small bowel bacterial overgrowth, cholestasis, side-effects of drug therapy, and liver disease-related metabolic disturbances play a role here [3,4,5,6,7]. Furthermore, liver cirrhosis can also induce other muscle changes like myopathy and/or myositis [8].

Previous investigations have suggested that sarcopenia is one of the factors predicting disease outcome in liver cirrhosis [9,10,11,12]. It has been shown that sarcopenia is associated with lower survival and quality of life, increases risk of complications like infections and encephalopathy, and lower post liver transplant survival in liver cirrhosis [3,9,10,11,12]. Therefore, early diagnosis of sarcopenia in liver cirrhosis is important. 

Several radiological investigations were proposed to identify and quantify muscle loss [13]. For example, several parameters of body composition, such as total fat area and/or muscle area can be estimated using computed tomography (CT) [13,14,15]. Furthermore, CT can also provide information on muscle fat degeneration based on tissue density [15]. Magnetic resonance imaging (MRI) using chemical shift encoding-based water-fat technique can also detect and quantify inter-and intramuscular adipose tissue fraction [16,17]. 

Of late, another imaging technique, namely diffusion weighted imaging (DWI), has been used in clinical practice. DWI is an established method, which offers diagnostic potential as well as additional information regarding composition and architecture of investigated tissue [18]. According to the literature, DWI quantified by apparent diffusion coefficient (ADC) can reflect different pathological changes, such as cell density, extracellular matrix, nucleic areas, and membrane permeability [19,20,21,22]. In addition, recent reports have highlighted the role of DWI in diagnosis of different muscle disorders [23,24,25]. However, this technique was not analyzed previously in detection of muscle changes in liver cirrhosis.

Presumably, DWI might better reflect muscle changes in liver cirrhosis than CT and/or conventional MR sequences. Furthermore, ADC might also be associated with liver function; for example, with the Model for End Stage-Liver Disease (MELD)-score. The MELD score is a widely used tool to assess disease severity for patients with end-stage liver disease awaiting liver transplantation [26,27].

The purpose of this study was to analyze ADC values of the skeletal musculature in patients with alcoholic liver cirrhosis and to compare them with those of healthy persons. Furthermore, we aimed to correlate muscle ADC values in liver cirrhosis with the MELD score.

## 2. Methods

This retrospective study was approved by the institutional ethics committee of the University of Leipzig (No. 356-10-13122010). Due to this retrospective, non-interventional, observational study design, patients’ informed consent was not required. 

### 2.1. Patients

**Figure 1:**
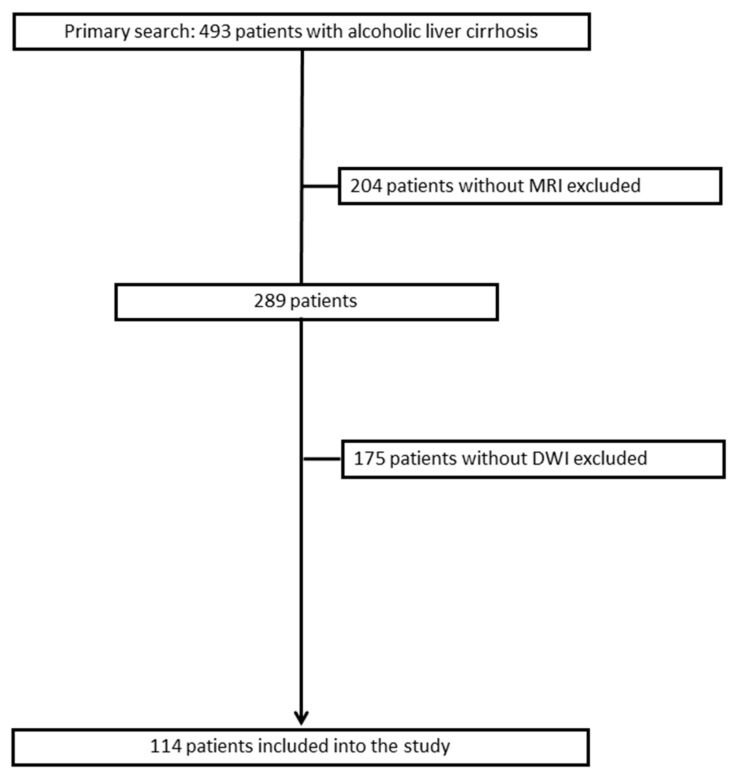
Flowchart of the data acquisition for the present study.

For this retrospective study, the databank of the radiological department was screened for patients with alcoholic liver cirrhosis, who were listed for liver transplantation and investigated by abdominal MRI including DWI (Figure 1). Patients with previous liver transplantation were excluded from the study. Overall, 114 patients were identified. There were 22 women and 92 men with a mean age of 56.5 ± 9.0 years: mean age 57.5 years.

In all patients, MELD score was calculated according to the previous descriptions [26,27] as follows:

MELD = 3.78 × ln[serum bilirubin (mg/dL)] + 11.2 × ln[international normalized ratio] + 9.57 × ln[serum creatinine (mg/dL)] + 6.43. 

Furthermore, in all patients, the following serological parameters were measured: C-reactive protein (CRP), lactate dehydrogenase (LDH), alanine aminotransferase (ALAT), and aspartate aminotransferase (ASAT).

Additionally, 12 healthy persons (5 women, 7 men), mean age, 42.1 ± 16.2 years, were prospectively investigated by MRI as a control group in the present study.

### 2.2. Diffusion Weighted Imaging and ADC Measurement

In all cases, MRI of the liver/trunk was performed using different 3T scanners. MRI protocol included the following sequences: a T2 weighted (T2w) fat-suppressed short tau inversion recovery (STIR) sequence, a half-Fourier acquisition single-shot turbo spin-echo (HASTE) sequence, T1 weighted (T1w) spin-echo (SE) images prior and after intravenous application of contrast medium as a dynamic measure. 

Diffusion weighted (DW) images were obtained with a multi-shot SE-EPI sequence (repetition time: 7200 ms; echo time: 50 ms; slice thickness: 5 mm; matrix: 88 × 134; field of view: 450 mm). DW imaging parameters included in all cases b values of 50, 400 and 800 s/mm².

**Figure 2:**
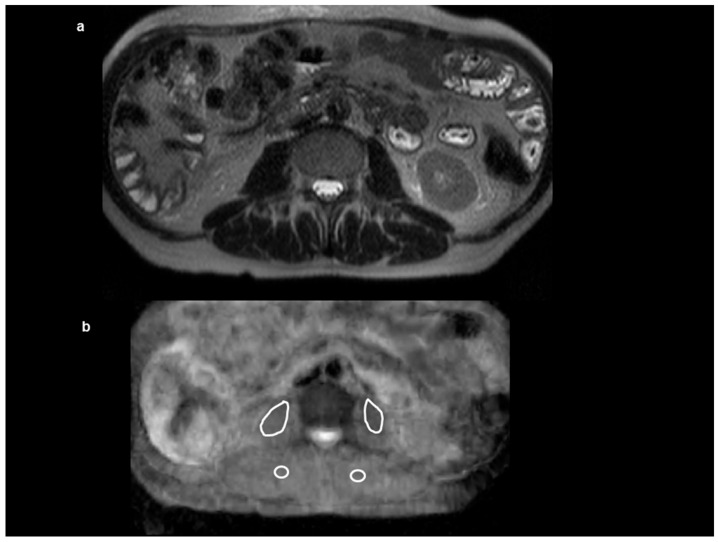
Anatomical sequence T2 weighted (T2w) at the level L2/3 (**a**). Apparent diffusion coefficient map (**b**) at the same level with the regions of interest within the skeletal musculature.

Apparent diffusion coefficient (ADC) maps were automatically generated by the implemented software. In all cases, polygonal regions of interest (ROI) were manually drawn on the ADC maps along the contours of the iliopsoas and paravertebral muscles (Figure 2). ROIs were placed to avoid fat areas and vessels. In every case, a cumulative mean ADC value of the skeletal musculature was estimated.

### 2.3. Statistical Analysis

Continuous variables were described by mean value, median, and standard deviation. Categorical variables were given as relative frequencies. The comparison of ADC values in groups was performed by ANOVA post hoc tests where the *p*-values are adjusted for multiple testing (Bonferroni correction). The association between ADC and MELD score was calculated by Spearman’s rank correlation coefficient.

## 3. Results

**Figure 3:**
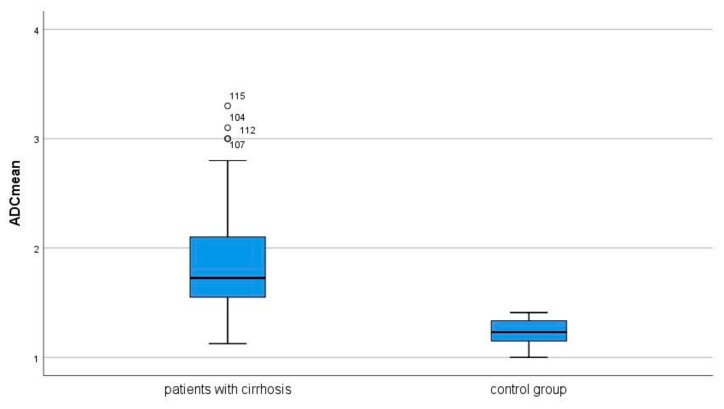
Apparent diffusion coefficient (ADC) values of the skeletal musculature in patients with liver cirrhosis and control group. Patients with liver cirrhosis show higher ADC values in comparison to the control group (1.85 ± 0.46 × 10^−3^ mm^2^ s^−1^ vs. 1.23 ± 0.12 × 10^−3^ mm^2^ s^−1^, *p* = 0.001).

The estimated ADC values of the skeletal musculature ranged from 1.13 to 3.3 × 10^−3^ mm^2^ s^−1^ with a median value of 1.73 × 10^−3^ mm^2^ s^−1^. The mean value was 1.85 ± 0.46 × 10^−3^ mm^2^ s^−1^. It was statistically significantly higher in comparison to those in the control group (*p* = 0.001) (Figure 3).

**Figure 4:**
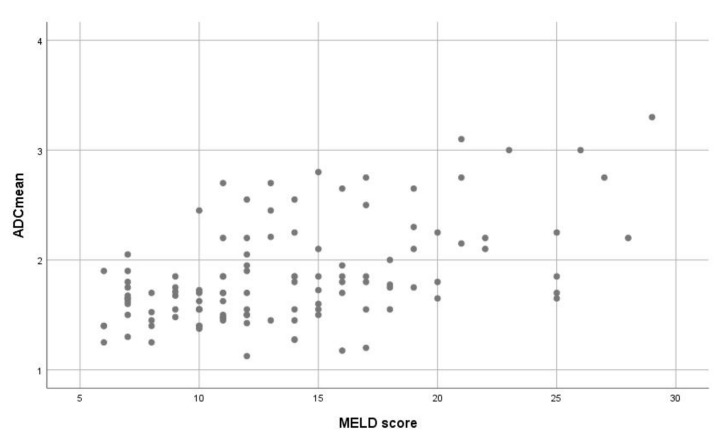
Associations between apparent diffusion coefficient values and the Model for End Stage-Liver Disease (MELD) score. The calculated correlation coefficient is 0.473, *p* = 0.0001.

The mean value of the MELD score was 13.7 ± 5.4, median value, 12, range, 6–29. ADC values correlated statistically significant with the MELD score (*r* = 0.473, *p* = 0.0001) (Figure 4). Furthermore, ADC values also differed between the subgroups with different values of the MELD score (Table 1).

ADC values correlated slightly with LDH (*r* = 0.381, *p* = 0.0001) and tended to correlate with CRP (*r* = 0.171, *p* = 0.07) and ALAT (*r* = −0.167, *p* = 0.076). There was no significant correlation between ADC and ASAT (*r* = 0.015, *p* = 0.870).

MELD score correlated statistically significant with LDH (*r* = 0.351, *p* = 0.0001), CRP (*r* = 0.284, *p* = 0.002) and ASAT (*r* = 0.283, *p* = 0.002).

## 4. Discussion

To the best of our knowledge, this is the first report about associations between ADC of the skeletal musculature in patients with liver cirrhosis.

Diffusion weighted imaging (DWI) is a magnetic resonance imaging technique based on measure of water diffusion in tissues [18]. Furthermore, water diffusion can be quantified by apparent diffusion coefficient [18]. Numerous studies investigated DWI and ADC findings in biomedicine. Thus, ADC has been reported as an essential imaging biomarker in different disorders, especially in oncology [19,20]. According to the literature, malignant and benign lesions show different ADC values [28]. Typically, malignant tumors have lower values in comparison to benign lesions [28]. Moreover, it has been shown that ADC correlated inversely with cell density in several malignant and benign diseases, and, therefore, can be used as a surrogate cellularity marker [19,29]. Furthermore, ADC can also reflect proliferation activity of different lesions measured by Ki 67 index [20]. In addition, in different muscle disorders, ADC can reflect pathological changes. For example, it has been shown that myositis and myopathy had statistically significant higher ADC values in comparison to unaffected muscles [23]. This phenomenon can be explained by the fact that inflammation induces degradation of muscle fibers and this facilitates water diffusion within muscles and, therefore, increases ADC values. Otherwise, muscle inflammation is associated with muscle edema, i.e. water collection between muscle fibers, and this factor results in increasing ADC values [23,24]. Moreover, ADC is correlated strongly with duration of motor unit action potential in myositis and, therefore, can be used as a quantitative biomarker of muscle alteration [25]. Furthermore, Holl et al. showed an increasing of ADC values of skeletal muscles after denervation [30]. In addition, muscle ischemia increased intramuscular ADC [31]. ADC is also able to reflect fine physiological changes of skeletal muscles. Thus, Yanagisawa et al. found that ADC significantly increased with exercise and decreased with cooling [32]. Other authors also observed a significant increase of ADC values after muscle exercise [33]. This finding may be related to increasing of perfusion and vascular spaces during exercise [33]. 

Overall, the reported data suggest that ADC can quantitatively estimate several physiological and pathological processes of the skeletal musculature.

We hypothesized that ADC can also be associated with muscle changes in liver cirrhosis. Our results confirmed this hypothesis. In fact, as shown, ADC values were statistically significant with the MELD score, namely increase of the MELD score was associated with an increase of ADC values of the skeletal musculature. This finding is very interesting and indicated that changes of muscle issue in cirrhosis may be similar with those in myopathy/myositis, which are also associated with an increase in muscle ADC values. This assumption is in agreement with the findings of Kilgour et al., who observed associations between muscle size and several immunological parameters like interleukin 6 [34]. Similar results were identified by Pin et al., who also found significant associations between muscle catabolism and interleukin 6 level [35]. Furthermore, Westbury et al. showed significant relationships between C-reactive protein and lower grip strength [36]. Additionally, a higher interleukin 8 level was associated with an increased risk of sarcopenia [36]. Moreover, the present study showed that ADC was also associated with LDH levels. This finding also supports our hypothesis of myopathy in liver cirrhosis. It is well known that LDH is elevated in different myopathies and can be used as a marker for muscle alteration [37]. 

As mentioned above, previously, only associations between muscle density on CT and/or muscle areas/diameter and the MELD score were analyzed. Thus, Kalafateli et al. did not find relationships between muscle density and the MELD score in patients after liver transplantation [5]. In the study by Englesbe et al., only a weak relationship was noted between preoperative MELD score and psoas muscle area [38]. These negative results may be related to a low sensitivity of CT and/or conventional MRI in detection of muscle changes. In fact, muscle fatty degeneration and decrease of muscle mass measured by CT occur predominantly in advanced cirrhosis [13,16]. In fact, in a study by Benjamin et al., the prevalence of sarcopenia identified on CT measures was 12.8% in patients with alcoholic liver cirrhosis [39]. Furthermore, the authors observed significant differences regarding muscle area and muscle index between healthy persons and patients with alcoholic liver cirrhosis [39]. However, there were no differences of muscle parameters between healthy and patients with compensated liver cirrhosis [39]. In the present study, ADC values of the patients with liver cirrhosis were statistically much higher than those in the control group, also in patients with lower MELD score values. This indicated that ADC is more sensitive in reflection of muscle changes in cirrhosis than previously used radiological methods.

Our findings are very important. Firstly, DWI is a standard sequence in the diagnosis of liver disorders. Therefore, ADC values of the skeletal musculature can be obtained without additional investigations/costs in these patients. Secondly, in contrast to CT, this technique is not associated with radiation burden. Thus, it can be repeated to control muscle changes under therapy. Finally, according to the literature, ADC measure has an excellent inter-reader agreement and test-retest-repeatability [40]. Therefore, ADC values can also be used as a biomarker in clinical routine.

The present study has several limitations. Firstly, a relatively small cohort of patients from a single center was analyzed. Secondly, this is a retrospective study. Clearly, further prospective studies with more patients are needed to confirm our preliminary results. Thirdly, further investigations should analyze associations of ADC with muscle specific parameters like myoglobin and creatine kinase in liver cirrhosis. 

## 5. Conclusions

ADC can reflect muscle changes in liver cirrhosis and it shows a statistically significant correlation with the MELD score. Therefore, ADC can be used as an imaging biomarker of myopathic changes in liver cirrhosis.

## Figures and Tables

**Table 1:** Comparison of apparent diffusion coefficient (ADC) values in dependency of Model for End Stage-Liver Disease (MELD) score.

MELD Score 0–9	MELD Score 10–19	MELD Score > 20
1.61 ± 0.21 × 10^−^^3^ mm^2^ s^−^^1^ *p* = 0.055 vs. MELD score 10–19; *p* = 0.001 vs. MELD score > 20	1.83 ± 0.42 × 10^−^^3^ mm^2^ s^−^^1^ *p* = 0.001 vs. MELD score > 20	2.34 ± 0.54 × 10^−^^3^ mm^2^ s^−^^1^
